# Assessing the role of Australia’s Pharmaceutical Benefits Scheme as a tool for addressing inequality in access to medications and allocation of public funds for pregnant women

**DOI:** 10.1136/bmjgh-2024-018565

**Published:** 2025-11-19

**Authors:** Hannah Jackson, Luke Grzeskowiak, Joanne Enticott, Sarah Wise, Emily J Callander

**Affiliations:** 1 School of Public Health, Faculty of Health, University of Technology Sydney, Ultimo, New South Wales, Australia; 2 College of Medicine and Public Health, Flinders University, Adelaide, South Australia, Australia; 3 South Australian Health and Medical Research Institute Limited, Adelaide, South Australia, Australia; 4 Monash Centre for Health Research and Implementation (MCHRI), Monash University, Clayton, Victoria, Australia

**Keywords:** Health systems evaluation, Maternal health, Public Health, Health Services Accessibility, Universal Health Care

## Abstract

**Introduction:**

Medication use during pregnancy is common, and socioeconomic disparities in access may contribute to maternal and fetal health inequalities. This study examines socioeconomic disparities in access to and expenditure on medications dispensed through Australia’s Pharmaceutical Benefits Scheme (PBS), evaluating its role in promoting equal access to medications for pregnant women.

**Methods:**

We analysed the Maternity1000 linked administrative dataset, which includes data on 57 443 women who gave birth in Queensland, Australia, between 1 July 2017 and 30 June 2018. Socioeconomic quintiles were assigned using the Index of Relative Socioeconomic Disadvantage. Medication prevalence rates, usage proportions and costs (2022/2023 Australian dollar) were calculated, followed by concentration curves and indices to assess inequality.

**Results:**

Medication prevalence was higher among more disadvantaged women (Q1 (most disadvantaged): 67% vs Q5 (least disadvantaged): 60%), who were also dispensed a higher average number of medications per pregnancy (Q1: 2.8 (95% CI 2.7 to 2.9) vs Q5: 2.4 (95% CI 2.3 to 2.5)). However, the total medication cost (patient contribution amount plus public subsidy) was, on average, lower for these women (Q1: $45 (95% CI 43 to 46) vs Q5: $52 (95% CI 50 to 54)), indicating potential disparities in access to newer, higher cost treatments. The unadjusted concentration index suggested mild pro-poor inequality in access (CI=−0.031; p<0.001), which was attenuated and statistically insignificant after adjusting for maternal demographic and clinical characteristics (CI_NA_=−0.007; p=0.089). Government expenditure on medications showed no significant socioeconomic inequality (unadjusted CI=0.001; p=0.965).

**Conclusion:**

The PBS facilitates equitable access to publicly funded medications for pregnant women. However, the uniform distribution of public funds across socioeconomic groups suggests possible limitations in progressivity, as public subsidies are not disproportionately benefiting the most disadvantaged women overall. This may reflect missed opportunities to distribute public funds more effectively and efficiently, particularly if disadvantaged women are under-represented in access to newer, higher cost therapies, and warrants ongoing evaluation.

**WHAT IS ALREADY KNOWN ON THIS TOPIC**

- Pregnant women are a marginalised population in terms of access to both newly developed and existing medications.
- Despite numerous barriers to access, the prevalence of medication use during pregnancy is both high and increasing, and a policy tool that facilitates equitable access to medications is necessary.
- The presence of a socioeconomic gradient in access to medication during pregnancy could further exacerbate the complex challenges disadvantaged women face in achieving the highest possible standard of maternal health outcomes.

**WHAT THIS STUDY ADDS**

- This study shows that the Pharmaceutical Benefits Scheme (PBS) is a valuable policy tool that promotes equitable distribution of publicly funded medications to pregnant women across the socioeconomic spectrum.
- Mild socioeconomic disparities in medication access were observed (pro-poor), which appeared to be driven by differences in maternal demographic and clinical characteristics.
- Government expenditure on medications was equally distributed across all levels of socioeconomic disadvantage, suggesting potential limitations in progressivity as the most disadvantaged women may not be receiving a proportionately greater share of public support.
- In a progressive system, we would expect a pro-poor distribution of public funding relative to socioeconomic disadvantage and health needs.
- Women in the most disadvantaged areas tended to access medications that were, on average, lower in total costs (ie, patient contribution amount plus public subsidy), suggesting potential disparities in access to newer, higher cost treatments.

**HOW THIS STUDY MIGHT AFFECT RESEARCH, PRACTICE OR POLICY**

- Australia’s PBS is an example of a public health policy tool that can promote equitable access to medications for all pregnant women.
- Continued evaluation of its capacity to support those with greater needs—especially within high-cost and high-volume areas of maternity care—is essential to ensure the PBS remains an effective equity tool.
- This study also highlights the need for enhancing regulatory pathways that facilitate PBS listing of pregnancy-relevant medications to enhance universal access to all essential pregnancy medications.

## Introduction

 Access to safe, effective and affordable medications during pregnancy is essential for managing both pre-existing and pregnancy-related medical conditions, supporting the health and safety of the mother and her developing baby throughout pregnancy and beyond. Medication use during pregnancy is common worldwide, with studies indicating more than 80% of pregnant women take at least one medication during their pregnancy.^1^,^4^ Yet, despite this widespread use, pregnant women’s access to these medications varies significantly across the globe.^15^,^7^ In 2015, the Sustainable Development Goals established an international commitment to ensure every woman can attain the highest possible standard of health and well-being, including during pregnancy.^8 9^ Improving medication access for pregnant women has become a global priority,^10^,^13^ with government policy playing a critical role in ensuring equal access.

Prior research indicates that achieving equal distribution of medications among women of different socioeconomic profiles can be challenging during pregnancy.^14^,^17^ The prohibitive costs associated with some medications that are not publicly subsidised exacerbate this issue. When medication financing relies on private sources, some women forgo necessary medications or experience significant financial stress.^18^ Socioeconomic disparities in medication access during pregnancy could further exacerbate the challenges already faced by women from disadvantaged backgrounds, such as increased risks of stillbirth, preterm birth and fetal growth restriction.^19 20^ This underscores the importance of a progressive funding system that provides greater support to pregnant women with greater socioeconomic disadvantage and health needs, thereby ensuring equitable access to necessary medications across all socioeconomic backgrounds. Such systems help to ensure that public resources are allocated fairly and responsibly, maximising their impact on population health and supporting the long-term sustainability of the healthcare system in the face of emerging needs.^21^,^24^

Australia’s healthcare system is founded on the principle of equity and equal access to healthcare for all, regardless of financial capability. Central to this is the Pharmaceutical Benefits Scheme (PBS), an integral policy instrument that supports Australia’s National Medicines Policy (NMP).^25^ Established in 1948,^26^ the PBS supports the provision of universal access to essential medications by subsidising the cost of approved medications with patients making a small copayment. This approach to medication funding aims to ensure that essential medicines are accessible, reliable and affordable, ultimately improving health, social and economic outcomes for every individual.^25^ While the importance of a progressive funding system for medications is frequently acknowledged, there remains a gap in our understanding of how effectively such systems, like the PBS, address disparities in medication access among pregnant women in practice.

The primary aim of this study is to fill this gap by assessing how effective Australia’s PBS is at providing equal access to medications for all pregnant women. More specifically, we aim to:

1. Identify any disparities in access to and expenditure on PBS-listed medications dispensed to pregnant women across different levels of socioeconomic disadvantage.
2. Analyse how PBS-listed medications and associated public expenditure are distributed across pregnant women experiencing different degrees of socioeconomic disadvantage.
3. Evaluate whether any identified inequalities in access or expenditure were equitable.

The findings from this study could influence policy reforms and resource allocation decisions, potentially improving healthcare outcomes for pregnant women both in Australia and offering insights for other countries seeking to respond to global calls for expanding access to medications for pregnant women.

## Methods

### Australia’s PBS

The PBS primarily provides medications dispensed by community pharmacies to patients in the community; however, it can also provide medication subsidies for hospital outpatients, individuals on discharge from hospitals and to hospital inpatients in select circumstances. All patients who are eligible for Medicare, Australia’s universal healthcare system, can access PBS-subsidised medications. As of 1 January 2024, general patients pay a maximum of $31.60 for most PBS-subsidised medications, while concession card holders (those on a low income) pay a maximum of $7.70; the Australian government covers any remaining cost.^26^ Additionally, a PBS Safety Net threshold ($277.20 for concession patients; $1647.90 for general patients in 2024) provides financial protection for individuals or families facing significant medication expenses within a calendar year. The PBS is a key component of Australia’s progressive health financing system, with copayments playing a crucial role in funding approved medications and ensuring equitable access to essential medicines. Notably, copayments are means tested, with lower income individuals paying reduced fees or being exempt from payments altogether. This progressive approach to medication expenses helps to ensure that essential medicines are accessible to people across all socioeconomic groups.

### Study design and population

We conducted an observational study using a population-level linked administrative dataset, Maternity1000.^27^ We analysed births occurring in the 2017/2018 financial year—the most recent year within the dataset. The dataset contains information on 57 485 women who gave birth (including live births and stillbirths of ≥20 weeks’ gestation) in Queensland, Australia, between 1 July 2017 and 30 June 2018 and their infants born during this timeframe. Variables reported for women who gave birth in the 2017/2018 financial year encompass all measures of that variable incurred during pregnancy for a woman who gave birth during that period. We used routinely collected information from the Queensland Perinatal Data Collection and linked records for pregnant women and their infants born within the timeframe to PBS claims and cost records between 1 September 2016 and 30 June 2018. Births occurring in both public and private sectors have been incorporated in this analysis. We chose to exclude any dispensings of medications listed in Section 100 of the PBS under the In Vitro Fertilisation (IVF) programme,^28^ given these are high-cost medications specifically used to help achieve pregnancy. All other prescriptions dispensed under the funding provisions of the PBS — both under copayment (dispensings priced less than or equal to the patient copayment amount defined in the *National Health Act 1953*, with copayments indexed annually and published in the Schedule of Pharmaceutical Benefits) *and* PBS-subsidised medications, including prescriptions dispensed under the Closing The Gap (CTG) scheme (which reduces medication costs for Aboriginal and Torres Strait Islander patients with, or at risk of, chronic disease; eligible general patients pay the concessional rate, while concessional patients pay no copayment, although brand premiums may still apply) — have been included in this analysis. Births for which there was no known birth year were excluded (n=93). All costs have been adjusted for inflation using the Reserve Bank of Australia’s inflation calculator and are reported in constant prices (2022/2023 Australian dollar). For reference, 1 AUD = 0.65 USD; 0.48 GBP; 0.57 EUR (June 2025).

### Socioeconomic groupings

Socioeconomic position was classified using the postcode that women reported for their usual place of residence. Postcodes were mapped to the Australian Bureau of Statistics’ Index of Relative Socioeconomic Disadvantage (IRSD) 2016. The IRSD is defined within the Australian Bureau of Statistics’ Socio-Economic Indexes for Areas^29^ and is a measure of relative disadvantage that incorporates income, education, employment, housing and other miscellaneous variables into a multidimensional framework. The resulting score runs on a continuum from most disadvantaged (low score) to a relative lack of disadvantage (high score) and has been further classified into IRSD quintiles for this study. Pregnancies were excluded from the analysis if an IRSD could not be assigned (error in recorded postcode, n=31; postcode without an assigned IRSD, n=11). This resulted in data being available for analysis on 57 443 pregnancies.

### Defining pregnancy within the dataset

Access to a PBS-listed medication during pregnancy was defined as every individual dispensing of a PBS-listed medication to a pregnant woman at any stage between day 31 of pregnancy and the date of delivery. A key privacy protection mechanism used in this data linkage study is that only the month and the year are provided for the date of delivery (ie, the date of the month is not provided). Consequently, within the dataset, each delivery date has been assigned as the first of the given month and year. Day 1 of pregnancy is then calculated by deducting the number of weeks and days of gestation at birth from the date of delivery. Given the actual date of delivery could be up to 30 days later than the date of delivery recorded in the dataset, we excluded the first 30 days of pregnancy from our analyses to minimise misclassification of any medications not actually dispensed during pregnancy. See online supplemental appendix 1 for a diagrammatic representation of the variables used to determine a dispensing that occurred during pregnancy.

### Statistical analysis

SAS V.9.4 was used to conduct all statistical analyses. For this study, missing data were retained in the analysis and explicitly reported as ‘missing’ in the results. The ‘public subsidy’ for each PBS dispensing is represented by the benefit amount in the PBS dataset. The ‘patient contribution amount’ reflects the out-of-pocket cost paid by the patient, excluding any brand premium or therapeutic group premium. The total cost of each dispensing is calculated as the sum of the patient contribution amount plus the public subsidy. This costing approach aligns with the methodology used in the annual PBS Expenditure and Prescriptions Reports.^30 31^ Wald confidence limits were used to calculate 95% confidence limits for prevalence rates. Count and cost data were both assumed to follow normal distributions, with 95% confidence limits being reported for all mean values. Descriptive statistics were used to illustrate the prevalence of pregnant women being dispensed at least one PBS-listed medication, the quantity and cost burden of all medications dispensed during pregnancy, and the most frequently dispensed medications and medications that consumed the greatest amount of government expenditure for women who gave birth in the 2017/2018 financial year. Analyses were stratified according to quintiles of socioeconomic disadvantage. Kendall’s coefficient of concordance was used to assess whether the rankings for volume of medication dispensed and total government expenditure during the 2017/2018 financial year for women experiencing the greatest level of disadvantage (quintile 1) were significantly different from medications dispensed to women experiencing less socioeconomic disadvantage.

To measure the degree of inequality in the socioeconomic distribution of PBS-listed medications dispensed to pregnant women in terms of both quantity dispensed and distribution of public funds, we plotted concentration curves and calculated concentration indices (CI). A concentration curve plots the cumulative proportion of a health variable (y-axis) as a function of the cumulative proportion of the population, ranked by socioeconomic disadvantage; beginning with the most disadvantaged and ending with the least disadvantaged (x-axis).^32^ Absolute equality in the variable of interest across socioeconomic groupings is shown by a 45-degree line added to the concentration curve graph. If the health variable is more heavily distributed among the most disadvantaged socioeconomic groups, the concentration curve will lie above the line of equality. The higher (lower) the concentration curve is above (below) the line of equality, the more concentrated that health variable is among the most disadvantaged (least disadvantaged). The CI measures the degree of inequality in a specified health variable as twice the area between the concentration curve and the line of equality.^32^ The value of a CI is bound between −1 and +1, whereby concentration curves lying above the line of equality indicate a pro-poor distribution of the variable and equate to a negative value for CI. The CI was calculated using the convenient regression formula below:


$$2\sigma_{r}^{2}\left(\frac{h_{i}}{\mu }\right)=\alpha +\beta r_{i}+\varepsilon_{i}$$

where $\sigma_{r}^{2}$ is the variance of the fractional rank, *h_i_* is the health sector variable (quantity of medications accessed, public expenditure on medication), μ is the mean of the health sector variable, *r_i_* is the fractional rank of each individual (*i*) in the socioeconomic distribution, α is the intercept, $\varepsilon_{i}$ is the error term and β is the ordinary least squares regression estimate of the CI.^32^ For this study, individual patient-level data are available for each health variable (quantity of medications dispensed and government expenditure) and socioeconomic variable (IRSD ranking). The CIs were calculated using microdata to maintain the highest degree of detail possible. Concentration curves were plotted using grouped data (deciles of disadvantage) for the socioeconomic variable in the graphs. Elsewhere, data are presented using IRSD quintiles to enhance readability of the results.

### Sensitivity analysis

We included non-pregnancy-specific medications in our analysis; therefore, we expected there would be extremely high-cost medications dispensed only to a limited number of women. The inclusion of these medications has the potential to skew our cost analyses, so we investigate the influence of these medications by excluding them at three different cost cut-offs (>$2000, >$1000 or >$500 per dispensing) for the total cost of the medication (ie, patient contribution amount plus public subsidy). We then recalculated the mean number of medications dispensed per pregnancy and the average total cost (patient contribution amount plus public subsidy) per dispensing.

In addition, we calculated a need-adjusted, or standardised, concentration index (CI_NA_) using an indirect method of standardisation. The maternal demographic and clinical characteristics that were controlled for were maternal age, body mass index, pre-existing medical conditions, pregnancy complications, Indigenous status and smoking during pregnancy before 20 weeks’ gestation. In this part of our analysis, count and cost data were both analysed using a negative binomial regression model with a log link function to account for the overdispersion of data. We included this CI_NA_ in the sensitivity analysis rather than the primary analysis because although research has established an association between socioeconomic disadvantage and multimorbidity in the general population,^33^,^35^ there is less clarity in the applicability of this association to pregnant populations.^36 37^

## Results

### Demographics

Data were available for 57 443 pregnancies. Demographic characteristics for women included in this study are reported in table 1.

**Table 1:** Demographic characteristics of women included in the analysis

Maternal characteristics	Q1 (most disadvantaged)	Q2	Q3	Q4	Q5 (least disadvantaged)	Total
Total	10 263 (17.87%)	9173 (15.97%)	14 706 (25.60%)	14 634 (25.48%)	8667 (15.09%)	57 443 (100%)
Maternal age						
<20 years	450 (4.38%)	273 (2.98%)	328 (2.23%)	187 (1.28%)	53 (0.61%)	1291 (2.25%)
20 to <35 years	8105 (78.97%)	7011 (76.43%)	11 207 (76.21%)	10 508 (71.81%)	5588 (64.47%)	42 419 (73.85%)
>35 years	1708 (16.64%)	1889 (20.59%)	3171 (21.56%)	3939 (26.92%)	3026 (34.91%)	13 733 (23.91%)
Missing	0 (0.00%)	0 (0.00%)	0 (0.00%)	0 (0.00%)	0 (0.00%)	0 (0.00%)
Country of birth						
Australia	7796 (75.96%)	7059 (76.95%)	10 623 (72.24%)	9631 (65.81%)	5805 (66.98%)	40 914 (71.23%)
Other	2467 (24.04%)	2114 (23.05%)	4083 (27.76%)	5000 (34.17%)	2862 (33.02%)	16 526 (28.77%)
Missing	0 (0.00%)	0 (0.00%)	0 (0.00%)	3 (0.02%)	0 (0.00%)	3 (0.01%)
Indigenous status						
Yes	1127 (10.98%)	944 (10.29%)	811 (5.51%)	344 (2.35%)	143 (1.65%)	3369 (5.86%)
No	9136 (89.02%)	8229 (89.71%)	13 895 (94.49%)	14 290 (97.65%)	8524 (98.35%)	54 074 (94.14%)
Missing	0 (0.00%)	0 (0.00%)	0 (0.00%)	0 (0.00%)	0 (0.00%)	0 (0.00%)
Gravidity						
Not first pregnancy	7529 (73.36%)	6485 (70.70%)	10 201 (69.37%)	9931 (67.86%)	5643 (65.11%)	39 789 (69.27%)
First pregnancy	2734 (26.64%)	2688 (29.30%)	4505 (30.63%)	4703 (32.14%)	3024 (34.89%)	17 654 (30.73%)
Missing	0 (0.00%)	0 (0.00%)	0 (0.00%)	0 (0.00%)	0 (0.00%)	0 (0.00%)
Plurality, n (%)						
Multiple	140 (1.36%)	142 (1.55%)	218 (1.48%)	205 (1.40%)	148 (1.71%)	853 (1.48%)
Singleton	10 123 (98.64%)	9031 (98.45%)	14 488 (98.52%)	14 429 (98.60%)	8519 (98.29%)	56 590 (98.52%)
Missing	0 (0.00%)	0 (0.00%)	0 (0.00%)	0 (0.00%)	0 (0.00%)	0 (0.00%)
Smoking status Before 20 weeks						
Yes	1984 (19.33%)	1375 (14.99%)	1463 (9.95%)	881 (6.02%)	338 (3.90%)	6041 (10.52%)
No	8260 (80.48%)	7773 (84.74%)	13 220 (89.90%)	13 738 (93.88%)	8319 (95.98%)	51 310 (89.32%)
Missing	19 (0.19%)	25 (0.27%)	23 (0.16%)	15 (0.10%)	10 (0.12%)	92 (0.16%)
Medical conditions						
Yes	4151 (40.45%)	3430 (37.39%)	5581 (37.95%)	5225 (35.70%)	3694 (42.62%)	22 081 (38.44%)
No	6112 (59.55%)	5743 (62.61%)	9124 (62.04%)	9409 (64.30%)	4973 (57.38%)	35 361 (61.56%)
Missing	0 (0.00%)	0 (0.00%)	1 (0.01%)	0 (0.00%)	0 (0.00%)	1 (<0.01%)
Pregnancy complication						
Yes	7723 (75.25%)	6857 (74.75%)	11 212 (76.24%)	10 991 (75.11%)	6700 (77.30%)	43 483 (75.70%)
No	2540 (24.75%)	2316 (25.25%)	3493 (23.75%)	3642 (24.89%)	1967 (22.70%)	13 958 (24.30%)
Missing	0 (0.00%)	0 (0.00%)	1 (0.01%)	1 (0.01%)	0 (0.00%)	2 (<0.01%)
BMI category						
Underweight	560 (5.46%)	432 (4.71%)	689 (4.69%)	775 (5.30%)	491 (5.67%)	2947 (5.13%)
Healthy weight	4140 (40.34%)	4214 (45.94%)	6984 (47.49%)	8092 (55.30%)	5261 (60.70%)	28 691 (49.95%)
Overweight	2434 (23.72%)	2227 (24.28%)	3557 (24.19%)	3281 (22.42%)	1728 (19.94%)	13 227 (23.03%)
Obese	2981 (29.05%)	2176 (23.72%)	3260 (22.17%)	2388 (16.32%)	1089 (12.56%)	11 894 (20.71%)
Missing	148 (1.44%)	124 (1.35%)	216 (1.47%)	98 (0.67%)	98 (1.13%)	684 (1.19%)
Assisted conception						
Yes	337 (3.28%)	399 (4.35%)	747 (5.08%)	1014 (6.93%)	861 (9.93%)	3358 (5.85%)
No	9926 (96.72%)	8773 (95.64%)	13 959 (94.92%)	13 620 (93.07%)	7806 (90.07%)	54 084 (94.15%)
Missing	0 (0.00%)	1 (0.01%)	0 (0.00%)	0 (0.00%)	0 (0.00%)	1 (<0.01%)
Funding for hospital admission						
Public	8760 (85.36%)	7138 (77.82%)	10 807 (73.49%)	10 068 (68.80%)	4229 (48.79%)	41 002 (71.38%)
Private	1487 (14.49%)	2021 (22.03%)	3871 (26.32%)	4528 (30.94%)	4417 (50.96%)	16 324 (28.42%)
Missing	16 (0.16%)	14 (0.15%)	28 (0.19%)	38 (0.26%)	21 (0.24%)	117 (0.20%)

BMI, body mass index.

### Prevalence of ≥1 medication being dispensed during pregnancy

The highest prevalence of taking at least one medication during pregnancy was observed for women experiencing the highest degree of socioeconomic disadvantage (Q1: 67.30% (95% CI 66.39% to 68.21%)). Prevalence rates continued to decrease as degree of socioeconomic disadvantage reduced (see table 2).

**Table 2:** Quantity of medications dispensed to pregnant women according to socioeconomic quintile

IRSD quintile	Women who gave birth, n	Prescriptions dispensed, n	Prevalence of ≥1 PBS-listed medication	Mean number of dispensings per pregnancy (95% CI)
1 (most disadvantaged)	10 263	28 535	67.30% (66.39–68.21)	2.78 (2.69 to 2.87)
2	9173	22 849	64.14% (63.16–65.13)	2.49 (2.41 to 2.58)
3	14 706	36 391	63.65% (62.87–64.42)	2.47 (2.41 to 2.54)
4	14 634	33 944	60.57% (59.78–61.36)	2.32 (2.25 to 2.39)
5 (least disadvantaged)	8667	20 675	60.34% (59.31–61.37)	2.39 (2.30 to 2.47)
Total	57 443	142 394	63.10% (62.70–63.49)	2.48 (2.44 to 2.51)

IRSD, Index of Relative Socioeconomic Disadvantage; PBS, Pharmaceutical Benefits Scheme.

### Volume and cost burden of all medications dispensed during pregnancy

The average number of medications accessed per pregnancy was highest for women experiencing the greatest degree of socioeconomic disadvantage (Q1: 2.78 (95% CI 2.69 to 2.87), as shown in table 2). Conversely, women least likely to be dispensed medications during pregnancy were those experiencing lower levels of socioeconomic disadvantage (quintiles 4 and 5), as shown in table 2 (see online supplemental appendix 2 for graphical representation of results).

Mean patient contribution amount per dispensing was proportional to the degree of socioeconomic disadvantage, with women experiencing the highest degree of disadvantage (quintile 1) paying less per dispensing compared with those in quintile 5 (Q1: $13.50 (95% CI 13.38 to 13.63); Q5: $21.09 (95% CI 20.93 to 21.26)), as shown in table 3. This is influenced by the progressive funding structure of the PBS, with ceiling prices for out-of-pocket payments (copayments) being means tested and reductions in copayments for Aboriginal and Torres Strait Islander patients with chronic disease. Notably, Indigenous status was more heavily concentrated in quintiles 1 and 2 (see table 1). As expected, the proportion of concessional-priced prescriptions dispensed was much greater for women experiencing higher levels of socioeconomic disadvantage (Q1: 52.65% vs Q5: 12.74%). In our patient population, the influence of reduced prices due to patients reaching the PBS Safety Net is negligible, at only 0.51% of all dispensings.

**Table 3:** Cost of medications dispensed during pregnancy according to socioeconomic quintile, constant prices (2022/2023 Australian dollar)

IRSD quintile	Public subsidy		Patient contribution		Total costs (public subsidy+patient contribution)	
	Sum (total government expenditure)	Mean (public subsidy per dispensing)	Sum (total patient contributions)	Mean (patient contribution per dispensing)	Sum (total costs)	Mean (total cost per dispensing)
**Primary analysis (all PBS-listed medications dispensed excluding S100 IVF drugs)**						
1 (most disadvantaged)	$889 146.51	$31.16 (29.67–32.65)	$385 306.90	$13.50 (13.38–13.63)	$1 274 453.42	$44.66 (43.14–46.18)
2	$730 411.54	$31.97 (30.04–33.89)	$351 552.82	$15.39 (15.23–15.54)	$1 081 964.37	$47.35 (45.38–49.33)
3	$1 037 646.22	$28.51 (26.67–30.36)	$617 271.41	$16.96 (16.84–17.08)	$1 654 917.63	$45.48 (43.60–47.35)
4	$1 342 757.17	$39.56 (34.69–44.43)	$640 263.13	$18.86 (18.73–18.99)	$1 983 020.29	$58.42 (53.53–63.31)
5 (least disadvantaged)	$638 110.67	$30.86 (29.00–32.73)	$436 131.21	$21.09 (20.93–21.26)	$1 074 241.88	$51.96 (50.03–53.89)
Total	$4 638 072.12	$32.57 (31.22–33.93)	$2 430 525.47	$17.07 (17.01–17.13)	$7 068 597.59	$49.64 (48.28–51.00)

IRSD, Index of Relative Socioeconomic Disadvantage; PBS, Pharmaceutical Benefits Scheme.

Mean public subsidy per dispensing appeared to be relatively consistent across socioeconomic quintiles, except for quintile 4, where the average subsidy was strongly influenced by a small quantity of high-cost medications (see table 3 and online supplemental appendix 4). The total medication cost (ie, patient contribution amount plus public subsidy) per dispensing, however, was higher on average for women experiencing the least socioeconomic disadvantage compared with those experiencing the greatest degree of disadvantage. This trend remained when high-cost medications were excluded from the results in a sensitivity analysis, as shown in table 4 and online supplemental appendix 2.

**Table 4:** Sensitivity analysis showing variations in quantity and cost of dispensings to pregnant women according to socioeconomic quintile, constant prices (2022/2023 Australian dollar)

				Total costs (public subsidy+patient contribution)	
IRSD quintile	Women who gave birth, n	Prescriptions dispensed, n	Mean number of dispensings per pregnancy (95% CI)	Sum (total costs)	Mean (total cost per dispensing)
**4.1 Sensitivity analysis excluding medications with a total cost (public subsidy+patient contribution) >$2000 per dispensing**					
1 (most disadvantaged)	10 263	28 522	2.78 (2.69 to 2.87)	$1 231 892.69	$43.19 (42.03–44.35)
2	9173	22 835	2.49 (2.40 to 2.57)	$1 030 205.06	$45.12 (43.65–46.58)
3	14 706	36 360	2.47 (2.41 to 2.54)	$1 536 488.03	$42.26 (41.27–43.24)
4	14 634	33 915	2.32 (2.24 to 2.39)	$1 672 663.11	$49.32 (47.99–50.65)
5 (least disadvantaged)	8667	20 665	2.38 (2.30 to 2.47)	$1 043 383.88	$50.49 (48.80–52.18)
Total	57 443	142 297	2.48 (2.44 to 2.51)	$6 514 632.77	$45.78 (45.20–46.36)
**4.2 Sensitivity analysis excluding medications with a total cost (public subsidy+patient contribution) >$1000 per dispensing**					
1 (most disadvantaged)	10 263	28 471	2.77 (2.68 to 2.86)	$1 154 693.93	$40.56 (39.66–41.46)
2	9173	22 766	2.48 (2.40 to 2.57)	$928 193.88	$40.77 (39.73–41.81)
3	14 706	36 296	2.47 (2.40 to 2.53)	$1 445 526.10	$39.83 (39.05–40.61)
4	14 634	33 774	2.31 (2.24 to 2.38)	$1 460 593.94	$43.25 (42.38–44.11)
5 (least disadvantaged)	8667	20 591	2.38 (2.29 to 2.46)	$926 689.47	$45.00 (43.87–46.14)
Total	57 443	141 898	2.47 (2.44 to 2.51)	$5 915 697.32	$41.69 (41.28–42.10)
**4.3 Sensitivity analysis excluding medications with a total cost (public subsidy+patient contribution) >$500 per dispensing**					
1 (most disadvantaged)	10 263	28 413	2.77 (2.68 to 2.86)	$1 111 159.32	$39.11 (38.29–39.92)
2	9173	22 708	2.48 (2.39 to 2.56)	$886 731.30	$39.05 (38.11–39.99)
3	14 706	36 218	2.46 (2.40 to 2.53)	$1 391 778.85	$38.43 (37.71–39.14)
4	14 634	33 692	2.30 (2.23 to 2.37)	$1 405 158.26	$41.71 (40.91–42.50)
5 (least disadvantaged)	8667	20 546	2.37 (2.28 to 2.46)	$896 949.58	$43.66 (42.60–44.71)
Total	57 443	141 577	2.46 (2.43 to 2.50)	$5 691 777.31	$40.20 (39.83–40.58)

IRSD, Index of Relative Socioeconomic Disadvantage; PBS, Pharmaceutical Benefits Scheme.

### Medications that accounted for the highest volume and cost of dispensings

Online supplemental appendix 3 shows that metoclopramide, an antiemetic, was the most frequently dispensed medication across all socioeconomic quintiles; however, it made up a smaller percentage of all dispensings for women experiencing less socioeconomic disadvantage (Q1: 11.73% vs Q5: 8.76%). Kendall’s coefficient of concordance (W=0.838; p<0.001) shows that there is a significant agreement in rankings of most dispensed medications across the socioeconomic quintiles. Ferric carboxymaltose was the medication that cost the government the greatest amount across all socioeconomic rankings (see online supplemental appendix 4); however, it represented the largest proportion of public expenditure within quintile 5 (least disadvantaged women). Kendall’s coefficient of concordance (W=0.752; p<0.001) showed there is again a significant agreement in the rankings of medications that consumed the greatest amount of public funds in this patient population. The spread of medications was found to be relatively narrow across all levels of socioeconomic status, with the top 10 most frequently dispensed medications within each socioeconomic quintile being responsible for approximately 50% of all items dispensed to pregnant women within the 2017/2018 financial year.

### Inequality, concentration curves and CIs

**Figure 1:**
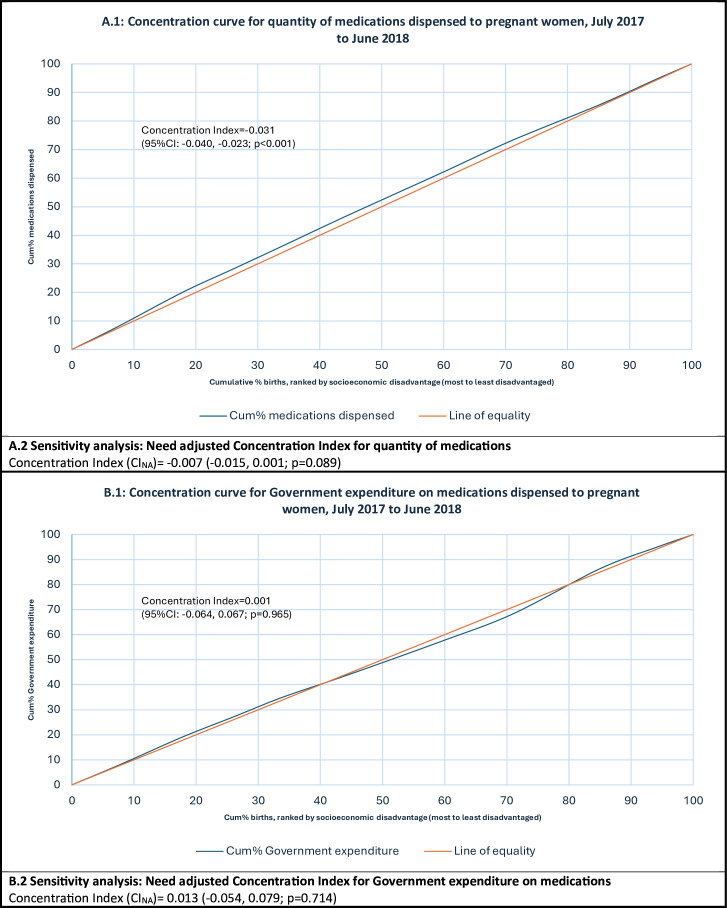
Concentration curve of access to PBS-funded prescription medications during pregnancy according to socioeconomic decile (IRSD). (A) Socioeconomic distribution of PBS-listed medication dispensings. (B) Socioeconomic distribution of total government expenditure. IRSD, Index of Relative Socioeconomic Disadvantage; PBS, Pharmaceutical Benefits Scheme.

In the context of this study, equality of access refers to pregnant women across socioeconomic quintiles receiving the same degree of access to medications; equality of government expenditure refers to total government expenditure on medications being dispersed equally across the socioeconomic quintiles. The distribution of medication dispensings in terms of volume of access was concentrated in women experiencing higher levels of disadvantage (ie, access is pro-poor), as shown by the concentration curve lying above the line of equality in figure 1A.1. The unadjusted CI of −0.031 (95% CI −0.040 to –0.023; p<0.001) confirms that access is somewhat unequal and is more concentrated among more disadvantaged women. The statistical significance of the pro-poor distribution appears to be accounted for by differing maternal characteristics among women in different socioeconomic quintiles. This is illustrated by a statistically insignificant pro-poor CI after adjusting for maternal demographic characteristics in CI_NA_=−0.007 (−0.015, 0.001; p=0.089). The distribution of government expenditure on medications dispensed to pregnant women showed that the distribution lies very close to the line of equality (as shown in figure 1B.1). The unadjusted CI calculation of 0.001 (95% CI −0.064 to 0.067; p=0.965) confirms that the distribution is not significantly different from the line of equality, and this observation persisted after adjustment for maternal characteristics (CI_NA_=0.013 (−0.054, 0.079; p=0.714)).

## Discussion

### Main findings

This study assessed the capacity of Australia’s main public policy tool for facilitating medication access, the PBS, in providing equal and equitable access to medications during pregnancy. Our findings indicate that women experiencing higher levels of socioeconomic disadvantage were more likely to be dispensed at least one PBS-listed medication during their pregnancy and were also more likely to be dispensed a higher number of medications per pregnancy. Conversely, women experiencing lower levels of socioeconomic disadvantage were dispensed fewer medications, but the medications they did access were generally more expensive in terms of total cost (patient contribution amount plus public subsidy). This trend persisted even after excluding high-cost medications dispensed in small quantities.

Our assessment of inequality in access to medications and associated public expenditure across the study population using concentration curves and indices reinforced the presence of a mild pro-poor pattern for the number of medications dispensed to pregnant women, with higher socioeconomic disadvantage being associated with increased access to medications within the PBS framework. This trend did not remain statistically significant after adjusting for differences in maternal characteristics, suggesting the pro-poor pattern observed was indeed equitable. Government expenditure on medications was evenly distributed across all levels of socioeconomic disadvantage among pregnant women, suggesting possible limitations in the system’s progressivity. Specifically, the most disadvantaged pregnant women did not receive a proportionately greater share of public funding, despite a likelihood of having greater health and economic needs. Additionally, women in the most disadvantaged areas tended to access medications that were, on average, lower in total costs (patient contribution amount plus public subsidy), indicating potential disparities in access to newer, more costly treatments. These findings warrant ongoing monitoring and further evaluation of therapeutic differences to ensure the PBS continues to function as an effective equity tool.

### Interpretation

Prior research in pregnant populations has focused on socioeconomic variations in healthcare utilisation,^38^ differences in the prevalence of maternal health conditions^39^ and socioeconomic disparities in maternal and fetal health outcomes.^19 38 40 41^ These studies consistently show that women of higher socioeconomic position tend to experience more favourable pregnancy outcomes,^41^ while those of low socioeconomic status face increased risks of adverse pregnancy outcomes.^19^ Our study adds to this body of work by evaluating how public resources, specifically medication access and government funding, are distributed within maternal healthcare systems. Internationally, it is commonly observed that socioeconomic disadvantage correlates with increased medication use, reflecting broader social gradients in health.^42 43^ Our results align with this pattern, revealing that within the framework of Australia’s PBS, medication access during pregnancy increases with socioeconomic disadvantage. This suggests that Australia’s PBS plays an important role in promoting equitable access to essential medications for pregnant women, including those facing socioeconomic disadvantage (as indicated and if PBS approved).

However, limitations in the system’s progressivity among pregnant populations warrant further evaluation. Although a mild pro-poor trend in medication access was observed, it appeared to be driven by differences in maternal health demographic and clinical characteristics, suggesting that access was equitable and driven by differences in need. Public funding for PBS medications was evenly distributed across socioeconomic groups, suggesting that public funding does not disproportionately benefit disadvantaged pregnant women overall. This was somewhat unexpected, given the progressive funding structure of the PBS, whereby patients with a greater ability to pay contribute more. In such a system, we would anticipate a pro-poor distribution of public subsidies, particularly for groups with greater medication needs (eg, low-income women, Indigenous Australians and those with pre-existing chronic conditions). PBS mechanisms like concessional copayments, CTG prescriptions and safety net thresholds are designed to reduce financial barriers to medication access for those most in need. When functioning effectively, we would expect the CI for public funding to be skewed towards disadvantaged groups. Our findings suggest that further investigation is needed to assess the capacity of the PBS to operate as a progressive funding system for pregnant women.

Importantly, our study also revealed an intriguing discrepancy: while the concentration curve showed an equal distribution of public funds across the socioeconomic spectrum, women experiencing greater socioeconomic disadvantage were accessing medications that were, on average, less expensive in terms of total medication cost (ie, patient contribution amount plus public subsidy). The inequality persisted even after excluding a small number of high-cost dispensings in sensitivity analyses. One interpretation is that less disadvantaged women may have greater access to newer, more expensive treatments. This trend aligns with prior observations, suggesting that privately funded obstetric care, often accessed by healthier and more affluent women, may be associated with higher average public subsidies for PBS-listed dispensings compared with publicly funded care.^44^ This discrepancy was present despite publicly funded maternity care services catering for a higher proportion of socioeconomically disadvantaged women.^45^ We recognise that inequality in access to higher cost medications coupled with lower dispensing rates among less disadvantaged women may have dampened the concentration curve and diluted the CI for the distribution of public funding, possibly obscuring inequalities that exist within specific therapeutic areas. Follow-up studies focused on high-cost and high-volume areas of obstetric care are essential to identify and address any existing disparities.

Other factors may also contribute to observed dispensing patterns and warrant further consideration. For example, pharmacy location may influence pricing as pharmacies can apply optional patient fees for under copayment dispensings,^46^ which may be more commonly applied in affluent areas. Additionally, variations in medical conditions, hospital funding mechanisms, prescriber knowledge and a lack of oversight of efficiency in private obstetric care may all play a role. Collectively, these complexities suggest that the observed disparities in medication costs and limitations in progressivity may not stem from the design of the PBS itself, but from broader systemic and structural barriers that constrain women’s access to pregnancy-relevant medications.

These findings must be interpreted within the broader context of Australia’s health policy landscape. The PBS is a dynamic policy tool that continues to evolve through regular updates to medication listings, pricing structures and access criteria. However, pregnant women continue to face significant upstream structural barriers to accessing medications through this scheme. These include their frequent exclusion from clinical trials, limited commercial incentives for developing pregnancy-relevant medications and ongoing shortages of obstetric medications.^47^ These challenges underscore the vulnerability of pregnant women to supply chain issues and highlight the need for ongoing assessment of the PBS’s capacity to promote equitable access to medications during pregnancy, particularly as the health policy environment evolves.

Notably, Australia’s NMP and Medicines Repurposing Program have undergone extensive review and reform since 2018,^25 48^ with the aim of improving equitable access to medications and supporting vulnerable populations. Nonetheless, no direct or measurable impact on pregnant women has yet been demonstrated. Some progress in more targeted access has been made, however, with PBS listing of higher cost medications like progesterone for pregnancy-related indications, which may influence patterns of access and public expenditure. Additionally, broader policy changes such as reduced patient copayments and 60-day dispensing for stable chronic conditions are also likely to affect distributive outcomes. Consequently, it is essential that similar investigations are conducted on an ongoing basis to monitor the effects of these policy changes. Importantly, studies such as this one provide a valuable comparison for evaluating the impact of evolving policy decisions on equitable access to medications during pregnancy.

### Limitations

This study has several limitations. First, due to delays in the release of administrative data, our analysis is based on data from the 2017/2018 financial year—the most recent year of data available for analysis. As a result, the findings may not reflect more recent updates to medication pricing or pharmaceutical policy. Second, our analysis included prescriptions dispensed under the CTG PBS Co-payment Program. Given that Indigenous women were more concentrated in disadvantaged areas, inclusion of CTG prescriptions may have increased the proportion of public subsidy relative to patient contributions. However, this is unlikely to have significantly influenced our overall conclusions, as public funding was found to be evenly distributed across socioeconomic groups. Third, concessional status was only available for women who had at least one PBS-listed item dispensed, limiting our ability to assess its impact on copayments. Fourth, socioeconomic disadvantage was assigned based on the mother’s residential postcode, which may not accurately reflect individual levels of disadvantage, introducing potential misclassification bias. Fifth, our analyses do not investigate the influence of pharmacy-level pricing variation, brand premiums, therapeutic group premiums or generic dispensings, all of which may impact the distribution of actual patient out-of-pocket expenses. These factors were excluded to maintain analytical focus and to align with the scope and conventions of existing PBS reporting structures. Sixth, our analysis specifically focused on medications accessed through the funding provisions of the PBS. We were unable to account for privately prescribed medications or those accessed through other funding avenues. The limited range of PBS-listed medications available for pregnant women reflects broader challenges, including a scarcity of clinical trial data, limited economic evidence necessary to expand the list of subsidised medications and limited industry incentives for pregnancy-relevant drug development. Finally, this study did not assess health outcomes associated with medication use during pregnancy. Consequently, we lack specific insight into the health outcomes that ensued (positive or negative) from pregnant women’s medication use. Despite these limitations, the PBS remains a valuable policy tool for promoting equitable access to medications during pregnancy and serves as a strong model for countries striving to enhance equitable medication access during pregnancy.

## Conclusion

In the global pursuit of equitable access to medication during pregnancy, addressing socioeconomic disparities remains a critical international concern. This study highlights Australia’s PBS as a strong example of a health policy mechanism that supports the equitable distribution of publicly subsidised medications to pregnant women across all socioeconomic groups. However, the relatively uniform allocation of public subsidies and lower total medication costs (comprising both the patient contribution and public subsidy) among disadvantaged women may suggest limitations in achieving true progressivity in access and financial protection. In a progressive system, we would typically expect a pro-poor distribution of public funding, whereby women facing greater socioeconomic disadvantage and health burden receive a greater share of public support.

Further research is needed to determine whether this pattern arises from the aggregated nature of the data or conceals disparities within specific therapeutic areas. Rather than signalling a shortcoming in the PBS itself, these findings may point to broader systemic and structural barriers that restrict pregnant women’s access to a wider range of pharmacotherapies; barriers that extend beyond the remit of the PBS alone. While our findings may indicate there are opportunities for ongoing improvement, the PBS remains a robust model for countries seeking to improve medication access during pregnancy, provided continuous evaluation and regulatory improvements are pursued to support pharmacoequity and strengthen maternal health outcomes.

## Supplementary material

10.1136/bmjgh-2024-018565 online supplemental file 1

## Data Availability

The datasets analysed for this study are not publicly available due to the strict ethics and privacy criteria that govern access to the data repository but are available from the corresponding author on appropriate request.
