# Supplementary material for: Assessing the role of Australia’s Pharmaceutical Benefits Scheme as a tool for addressing inequality in access to medications and allocation of public funds for pregnant women
Source: BMJ Glob Health. 2025 Nov 19;10(11):e018565. doi: 10.1136/bmjgh-2024-018565 (PMC12636929; doi:10.1136/bmjgh-2024-018565)
Supplement: online supplemental file 1 [file bmjgh-10-11-s001.pdf]

SUPPLEMENTARY MATERIAL

Assessing the role of Australia's Pharmaceutical Benefits Scheme as a tool for addressing inequality in access to medications and allocation of public funds for pregnant women.

Hannah Jackson[a], Luke Grzeskowiak[b,c], Joanne Enticott[d], Sarah Wise[a], Emily Callander[a *]

a. School of Public Health, Faculty of Health, University of Technology Sydney, New South Wales, Australia
b. College of Medicine and Public Health, Flinders University, South Australia, Australia
c. Women and Kids Theme, South Australian Health and Medical Research Institute, South Australia, Australia
d. Monash Centre for Health Research and Implementation (MCHRI), School of Public health and Preventive Medicine, Monash University, Clayton, Victoria, Australia

* *Corresponding author*: EC: emily.callander@uts.edu.au ORCID 0000-0001-7233-6804
Professor Emily Callander
School of Public Health, Faculty of Health
University of Technology Sydney
PO Box 123 Broadway NSW 2007 Australia

Co-authors: HJ: hannah.m.jackson@student.uts.edu.au ORCID 0000-0002-9788-8821
LG: luke.grzeskowiak@flinders.edu.au ORCID 0000-0001-8554-4696
JE: joanne.enticott@monash.edu ORCID 0000-0002-4480-5690
SW: sarah.wise@uts.edu.au ORCID 0000-0003-3513-3471

Table of Contents

Appendix 1: Diagram showing how dispensings occurring during pregnancy were calculated within the dataset 2

Appendix 2: Sensitivity analysis showing mean cost (Total cost = patient contribution amount plus public subsidy) per dispensing and mean number of medications dispensed per pregnancy, stratified according to socioeconomic disadvantage, 2017/18, in constant prices (AUD 2022/23). 3

Appendix 3: Top 10 most frequently dispensed drugs (quantity) for women who gave birth in 2017/18 fiscal year, according to socioeconomic ranking (IRSD quintile) 4

Appendix 4: Medications that consumed the greatest amount of public funds (i.e., Government expenditure) for women who gave birth in 2017/18 fiscal year, according to socioeconomic ranking (Top 10), constant prices (AUD 2022/23) 5

Appendix 1: Diagram showing how dispensings occurring during pregnancy were calculated within the dataset

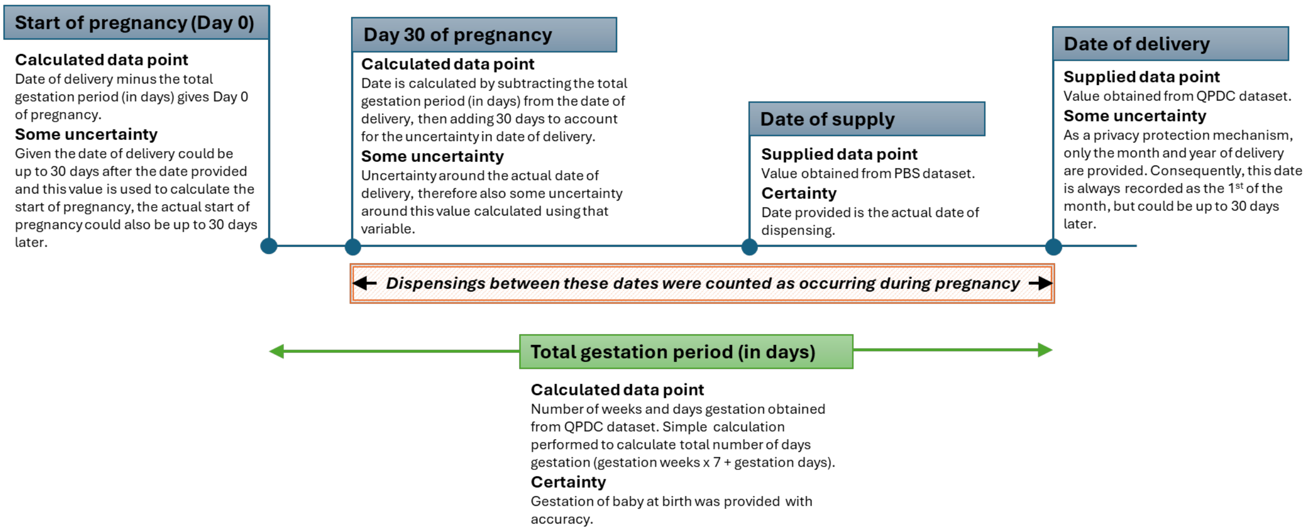

Appendix 2: Sensitivity analysis showing mean cost (Total cost = patient contribution amount plus public subsidy) per dispensing and mean number of medications dispensed per pregnancy, stratified according to socioeconomic disadvantage, 2017/18, in constant prices (AUD 2022/23).

A2.1 Total medication cost (Total = patient contribution amount + public subsidy) per dispensing

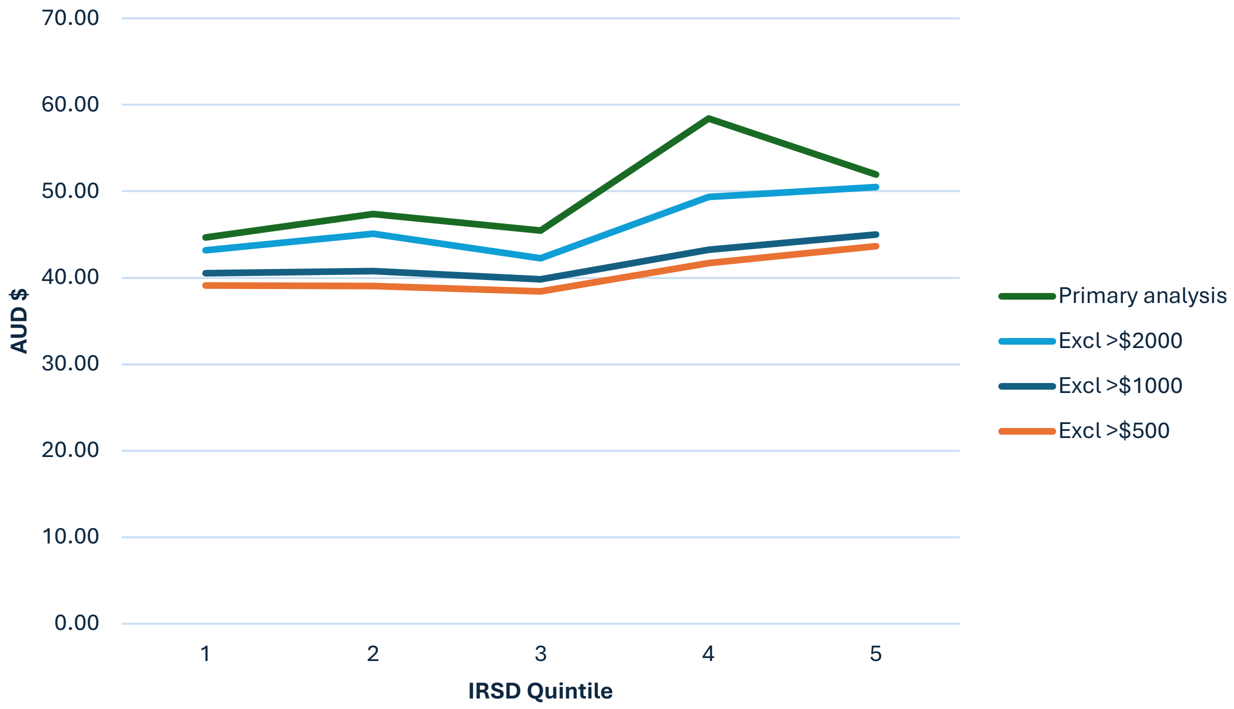

A2.2 Mean number of medications dispensed per pregnancy

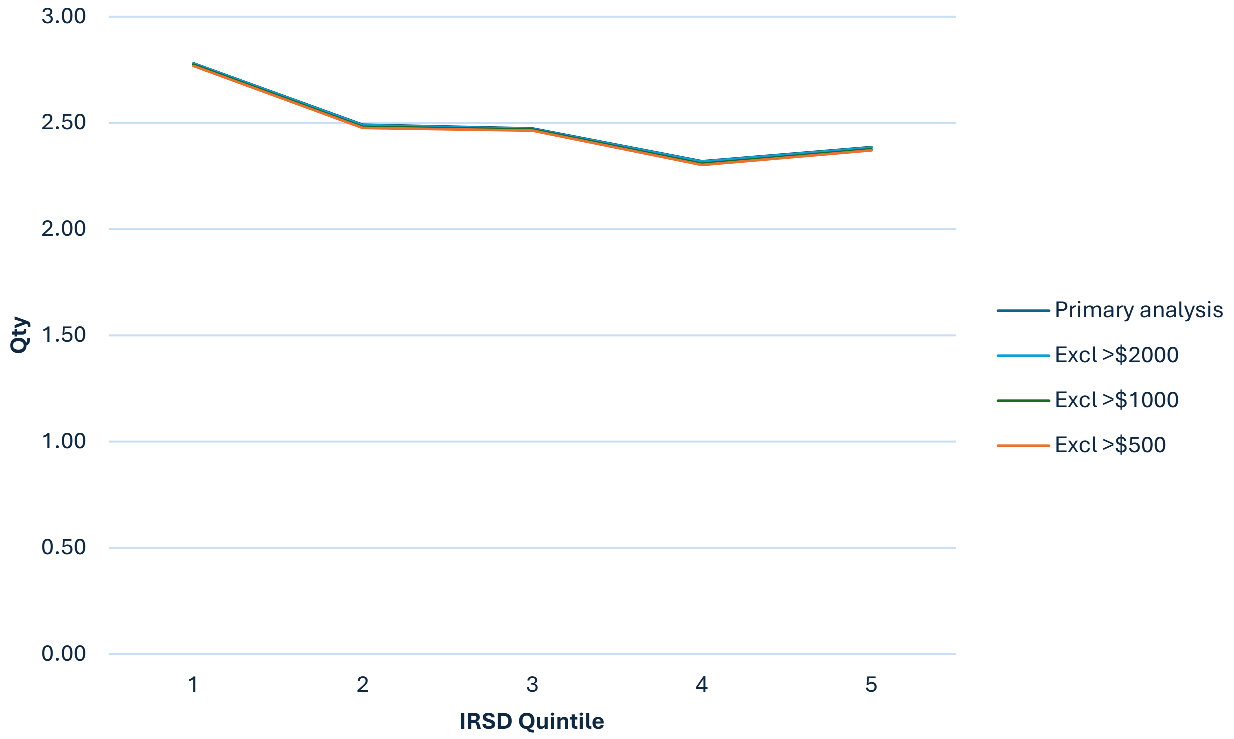

Appendix 3: Top 10 most frequently dispensed drugs (quantity) for women who gave birth in 2017/18 fiscal year, according to socioeconomic ranking (IRSD quintile)

INDIVIDUAL MEDICATIONS																			
Q1				Q2				Q3				Q4				Q5			
DRUG_NAME	N	%	Cum%	DRUG_NAME	N	%	Cum%	DRUG_NAME	N	%	Cum %	DRUG_NAME	N	%	Cum%	DRUG_NAME	N	%	Cum%
Metoclopramide	3348	11.73	11.73	Metoclopramide	2599	11.37	11.37	Metoclopramide	4182	11.49	11.49	Metoclopramide	3564	10.50	10.50	Metoclopramide	1812	8.76	8.76
Cefalexin	2774	9.72	21.45	Cefalexin	2316	10.14	21.51	Cefalexin	3205	8.81	20.30	Amoxicillin	2648	7.80	18.30	Amoxicillin	1458	7.05	15.82
Amoxicillin	2220	7.78	29.23	Amoxicillin	1743	7.63	29.14	Amoxicillin	3015	8.29	28.58	Cefalexin	2640	7.78	26.08	Cefalexin	1389	6.72	22.53
Metformin	1435	5.03	34.26	Metformin	1007	4.41	33.55	Metformin	1622	4.46	33.04	Sertraline	1306	3.85	29.93	Levothyroxine	972	4.70	27.24
Ondansetron	1071	3.75	38.02	Sertraline	877	3.84	37.38	Sertraline	1544	4.24	37.28	Metformin	1251	3.69	33.61	Sertraline	904	4.37	31.61
Sertraline	959	3.36	41.38	Ondansetron	811	3.55	40.93	Ranitidine	1255	3.45	40.73	Ranitidine	1219	3.59	37.20	Enoxaparin sodium	880	4.26	35.86
Paracetamol + codeine	955	3.35	44.72	Ranitidine	796	3.48	44.42	Ondansetron	1243	3.42	44.15	Enoxaparin sodium	1195	3.52	40.72	Metformin	880	4.26	40.12
Ranitidine	910	3.19	47.91	Paracetamol + codeine	735	3.22	47.63	Paracetamol + codeine	1154	3.17	47.32	Levothyroxine	1091	3.21	43.94	Ranitidine	743	3.59	43.71
Enoxaparin sodium	845	2.96	50.87	Enoxaparin sodium	642	2.81	50.44	Levothyroxine	1067	2.93	50.25	Ondansetron	1030	3.03	46.97	Rabeprazole	657	3.18	46.89
Salbutamol	818	2.87	53.74	Escitalopram	612	2.68	53.12	Enoxaparin sodium	996	2.74	52.99	Escitalopram	974	2.87	49.84	Ferric carboxymaltose	605	2.93	49.82
% refers to number of dispensings for a given medication as a proportion of total dispensings for all medications																			

Appendix 4: Medications that consumed the greatest amount of public funds (i.e., Government expenditure) for women who gave birth in 2017/18 fiscal year, according to socioeconomic ranking (Top 10), constant prices (AUD 2022/23)

INDIVIDUAL MEDICATIONS																								
Q1					Q2					Q3					Q4					Q5				
DRUG_NAME	N	Govsum	%	Cum %	DRUG_NAME	N	Govsum	%	Cum %	DRUG_NAME	N	Govsum	%	Cum %	DRUG_NAME	N	Govsum	%	Cum %	DRUG_NAME	N	Govsum	%	Cum %
Ferric carboxymaltose	573	$184465.69	20.75	20.75	Ferric carboxymaltose	545	$171642.17	23.50	23.50	Ferric carboxymaltose	820	$250083.01	24.10	24.10	Ferric carboxymaltose	884	$265169.91	19.75	19.75	Ferric carboxymaltose	605	$186759.7	29.27	29.27
Enoxaparin sodium	845	$121464.12	13.66	34.41	Enoxaparin sodium	642	$82637.53	11.31	34.81	Enoxaparin sodium	996	$122895.27	11.84	35.94	Ivacaftor	9	$237758.41	17.71	37.45	Enoxaparin sodium	880	$93718.33	14.69	43.95
Insulin isophane human	436	$55809.02	6.28	40.68	Adalimumab	23	$48016.13	6.57	41.39	Insulin isophane human	438	$52312.04	5.04	40.99	Enoxaparin sodium	1195	$138420.19	10.31	47.76	Adalimumab	22	$38645.14	6.06	50.01
Insulin aspart	240	$50162.00	5.64	46.33	Insulin aspart	175	$38140.05	5.22	46.61	Insulin aspart	279	$50995.18	4.91	45.90	Adalimumab	50	$82036.14	6.11	53.87	Mesalazine	128	$37489.11	5.88	55.89
Etanercept	22	$32984.73	3.71	50.04	Insulin isophane human	252	$36556.30	5.00	51.61	Mesalazine	123	$36193.79	3.49	49.39	Insulin isophane human	366	$52321.29	3.90	57.77	Natalizumab	15	$26244.48	4.11	60.00
Tenofovir disoproxil	28	$25622.15	2.88	52.92	Insulin glargine	68	$26717.65	3.66	55.27	Infliximab	12	$35554.05	3.43	52.82	Insulin aspart	206	$42559.08	3.17	60.94	Insulin aspart	123	$24744.70	3.88	63.88
Insulin glargine	54	$23511.57	2.64	55.56	Certolizumab pegol	16	$24659.82	3.38	58.65	Vedolizumab	8	$29204.03	2.81	55.63	Mesalazine	146	$41643.82	3.10	64.04	Insulin isophane human	153	$21287.87	3.34	67.21
Adalimumab	14	$22737.80	2.56	58.12	Infliximab	8	$23650.39	3.24	61.89	Insulin glargine	66	$27764.68	2.68	58.31	Infliximab	15	$35523.53	2.65	66.69	Infliximab	7	$19091.94	2.99	70.20
Insulin detemir	49	$18369.58	2.07	60.18	Etanercept	13	$17085.88	2.34	64.22	Etanercept	21	$27234.19	2.62	60.93	Etanercept	20	$27744.78	2.07	68.75	Insulin detemir	57	$18058.21	2.83	73.03
Mesalazine	51	$17764.76	2.00	62.18	Tenofovir disoproxil	18	$16265.12	2.23	66.45	This item has been suppressed due to privacy restrictions					Tenofovir disoproxil	24	$26186.80	1.95	70.70	Certolizumab pegol	10	$16918.31	2.65	75.69
